# Supplementary material for: Enhancement of the Local CD8+ T-Cellular Immune Response to Mycobacterium tuberculosis in BCG-Primed Mice after Intranasal Administration of Influenza Vector Vaccine Carrying TB10.4 and HspX Antigens
Source: Vaccines (Basel). 2021 Nov 3;9(11):1273. doi: 10.3390/vaccines9111273 (PMC8626046; doi:10.3390/vaccines9111273)
Supplement: Supplementary file 1 [file vaccines-09-01273-s001.zip › vaccines-1380702-supplementary.pdf]

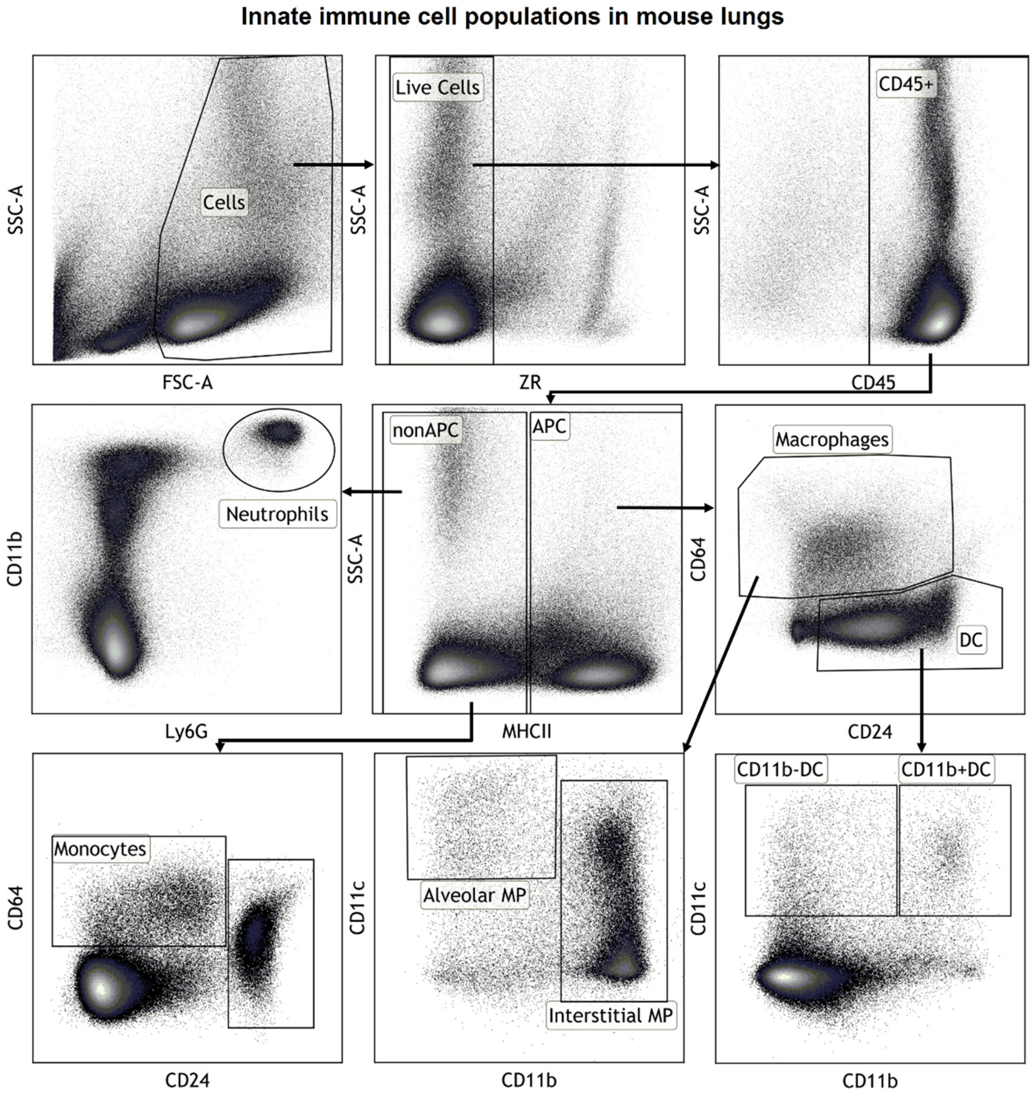

Figure S1. Gating strategy used to identify innate immunity cell subsets in the mouse lung. Cell doublets were eliminated using FSC-A/FSC-H light scattering (not shown). Live, single cell population was identified based on the FSC-A/SSC-A light scattering and binding of the Zombie Red dye. Immune cells were gated according to the presence of the CD45 marker. Sequential gating was used to identify antigen-presenting cells (APC, $CD45^{+}MHCII^{+}$) and non-APC cells ($CD45^{+}MHCII^{-}$), neutrophils ($CD45^{+}Ly6G^{+}CD11b^{+}$), moncytes ($MHCII^{-}CD64^{+}CD24^{+}$), alveolar macrophages (Alveolar MP, $MHCII^{+}CD64^{+}CD11c^{+}CD11b^{-}$), interstitial macrophages (Interstitial MP, $MHCII^{+}CD64^{+}CD11b^{+}CD11c^{+/-}$), $CD11b^{-}$ dendritic cells ($CD11b^{-}DC$, $CD45^{+}CD11c^{+}CD11b^{-}MHCII^{+}CD64^{-}CD24^{+}$) and $CD11b^{+}$ dendritic cells ($CD11b^{+}DC$, $CD45^{+}CD11c^{+}CD11b^{+}MHCII^{+}CD64^{-}CD24^{+}$).

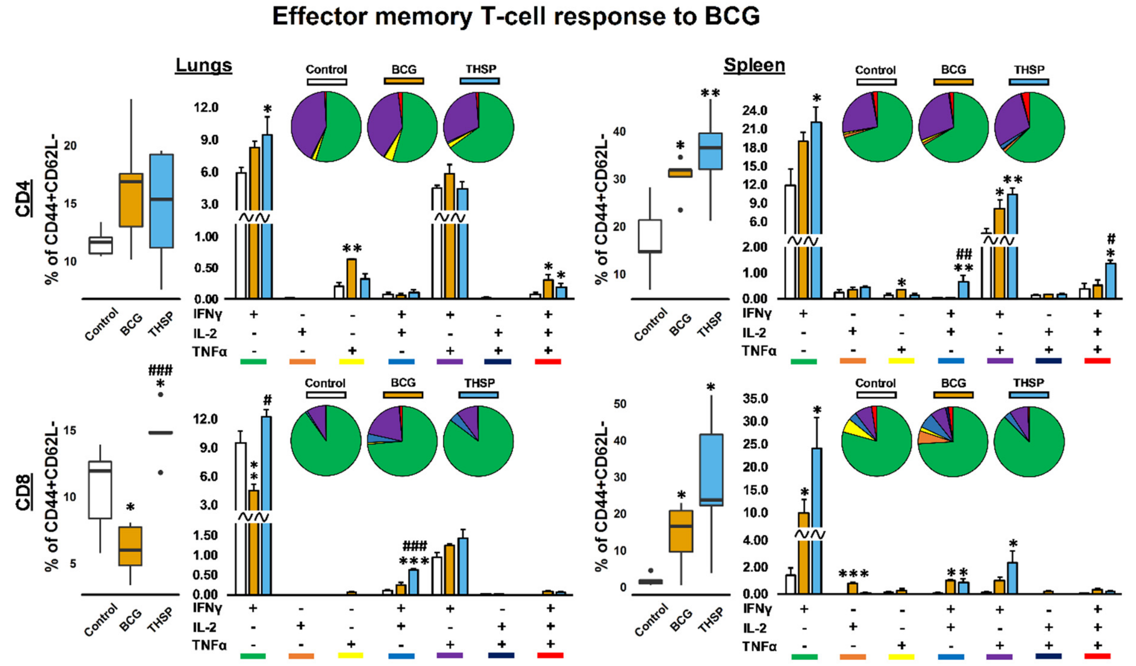

Figure S2. BCG-specific effector memory T-cellular immune response in lungs and spleen of boost-immunized mice, 5 weeks after *M. Tuberculosis* challenge. CD4/8$^+$CD44$^+$CD62L$^-$ effector memory (EM) T-cells from lungs and spleen expressing IFNγ, IL-2 and TNFα after 24 h stimulation with BCG. Box-plots represent total amount of cytokine-producing EM T-cells. Bar-plots represent the average percent of each cytokine-producing population within total EM T-cells subset (Mean ± SE). Pie-charts show the average percent of each cytokine-producing population within total subset of cytokine-producing cells. Differences between groups were analyzed using Student's t test. *p<0.05, **p<0.01, and ***p<0.001, n = 5 in comparison with control group; #p<0.05, ##p<0.01, and ###p<0.001, n = 5 compared with BCG.

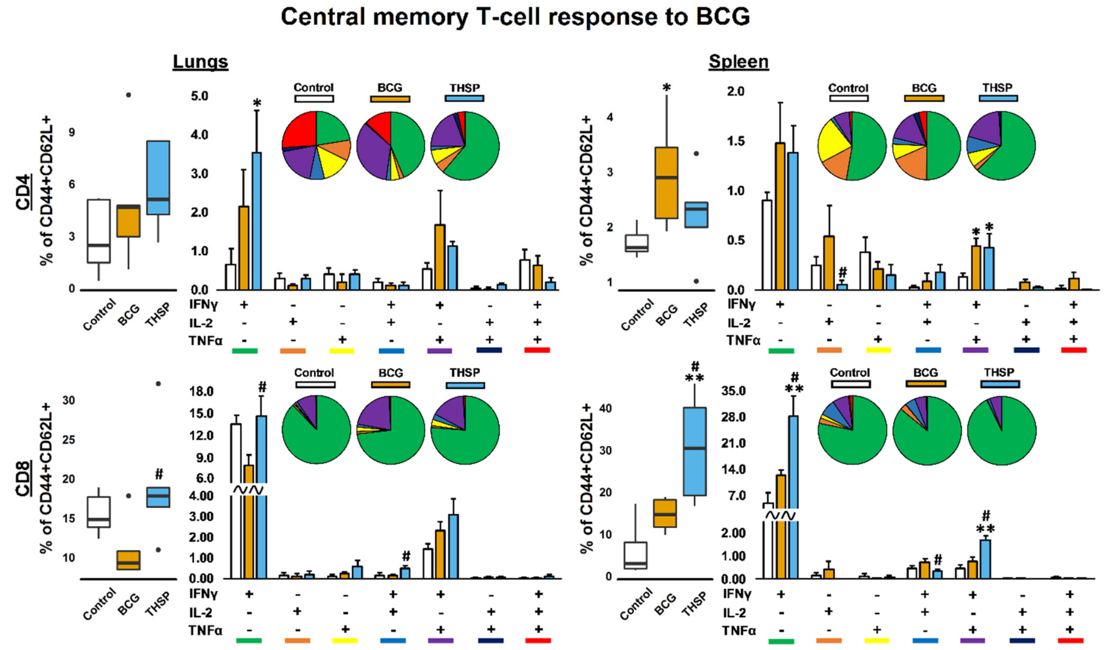

Figure S3. BCG-specific central memory T-cellular immune response in lungs and spleen of boost-immunized mice, 5 weeks after *M. Tuberculosis* challenge. CD4/8$^+$CD44$^+$CD62L$^-$ central memory (CM) T-cells from lungs and spleen expressing IFNγ, IL-2 and TNFα after 24 h stimulation with BCG. Box-plots represent total amount of cytokine-producing EM T-cells. Bar-plots represent the average percent of each cytokine-producing population within total CM T-cells subset (Mean ± SE). Pie-charts show the average percent of each cytokine-producing population within total subset of cytokine-producing cells. Differences between groups were analyzed using Student's t test. *p<0.05, **p<0.01, and ***p<0.001, n = 5 in comparison with control group; #p<0.05, ##p<0.01, and ###p<0.001, n = 5 compared with BCG.
